# QuickStats

**Published:** 2015-08-07

**Authors:** FROM THE NATIONAL CENTER FOR HEALTH STATISTICS

**Figure:**
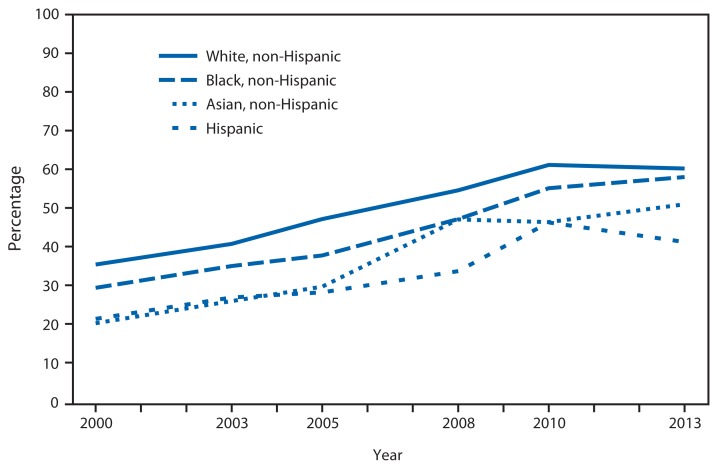
Colorectal Cancer Screening* Among Adults Aged 50–75 Years, by Race and Hispanic Origin^†^ — National Health Interview Survey,^§^ United States, 2000–2013 * Includes reports of home fecal occult blood test (FOBT) in the past year, sigmoidoscopy procedure in the past 5 years with FOBT in the past 3 years, or colonoscopy in the past 10 years, based on the most recent guidelines from the U.S. Preventive Services Task Force. Colorectal cancer tests and procedures are performed for diagnostic and screening purposes. ^†^ Categories of non-Hispanic persons are for respondents who selected one racial group; respondents had the option to select more than one racial group. Hispanic origin refers to persons who are of Hispanic ethnicity and might be of any race or combination of races. Non-Hispanic refers to all persons who are not of Hispanic ethnicity, regardless of race. ^§^ Estimates are based on household interviews of a sample of the civilian noninstitutionalized population. Questions about colorectal tests or procedures differed slightly on the National Health Interview Survey and were asked on an intermittent schedule in 2000, 2003, 2005, 2008, 2010, and 2013. Additional information available at http://www.cdc.gov/nchs/data/hus/hus14.pdf (page 404).

During 2000–2013, among adults aged 50–75 years, the use of colorectal cancer tests or procedures increased for all racial and ethnic groups shown. Non-Hispanic Asian adults had the largest increase; the percentage more than doubled from 20.6% in 2000 to 51.2% in 2013. Although increases were observed among all groups, in 2013 the prevalence of colorectal cancer screening remained higher among non-Hispanic white (60.4%) and non-Hispanic black (58.2%) adults and lower among non-Hispanic Asian (51.2%) and Hispanic (41.5%) adults.

**Source:** Health, United States, 2014 (with special feature on adults aged 55–64, Table 78). Available at http://www.cdc.gov/nchs/data/hus/hus14.pdf.

**Reported by:** Hashini Khajuria, MPA, hwq6@cdc.gov, 301-458-4253.

